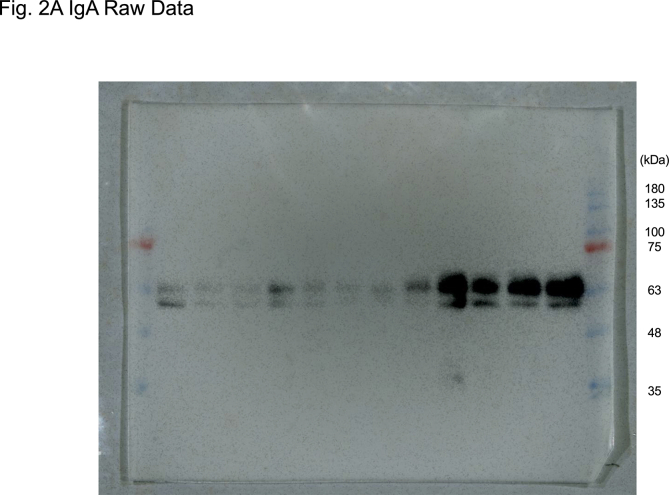# Intestinal antibody repertoire is altered by diabetes and varies depending on the pathogenesis

**DOI:** 10.1016/j.bbrep.2025.101964

**Published:** 2025-03-02

**Authors:** Miho Chikazawa, Ken-Ichiro Minato

**Affiliations:** Department of Applied Biological Chemistry, Faculty of Agriculture, Meijo University, 1-501 Shiogamaguchi, Nagoya, Japan

**Keywords:** Diabetes, IgA, Antibody repertoire, Intestinal immunity

## Abstract

Intestinal immunity is an important system for host defense and it is influenced by various factors such as diet and diseases. To elucidate the relationship between intestinal immunity and type 2 diabetes, we analyzed the effects of diabetes on intestinal antibody production and IgA repertoire using high-fat diet-fed mice and genetically diabetic KK-A^y^ mice model. The antibody level in the small intestine increased only in KK-A^y^ mice. We also confirmed that the IgA repertoire in both models experienced significant changes when compared to that in control mice, and no shared characteristics were observed between the two diabetic models. Antibody production in the intestine is influenced by stimuli associated with the onset of diabetes, and the types of induced IgA would differ depending on the process of disease onset.

## Highlights

• In KK-A^y^ mice, antibody production is increased in the intestine.
• In a diabetic mouse model, intestinal IgA repertoire changes significantly.
• Changes in intestinal IgA repertoire differ according to the type of model of diabetes.

## Abbreviations

HFD: high-fat diet
IgH: immunoglobulin heavy chain
IgK: immunoglobulin kappa light chain

## 1 Introduction

Antibodies possess diverse specificities, and highly specific antibodies against invading pathogens are important to prevent infection. In bodily fluids, a diverse population of antibodies comprises a cluster that is referred to as an “antibody repertoire” [1]. The antibody repertoire is influenced by several factors, including antigen exposure, disease, and diet. Changes in the antibody repertoire reflecting an individual's health status.

The gastrointestinal tract plays an important role in mucosal immunity as it harbors various immunological cells. Additionally, various molecules in the gastrointestinal tract, including food, bacteria, and secreted factors from tissues, may induce an active immune responses and alter antibody production [2,3]. Intestinal microbiota-derived short-chain fatty acids reportedly promote immunoglobulin A (IgA) production and impact IgA responsiveness to microbes in the intestine [4,5]. Furthermore, food compounds, such as lactic acid bacteria, lactoferrin, and glutamine, reportedly enhance intestinal IgA production [[6], [7], [8]].

Type 2 diabetes mellitus affects the immune system by increasing susceptibility to infections [9], which is associated to the onset and progression of the disease. Although changes in the intestinal environment that are associated with diabetes, such as inflammation and microbiota composition, is involved in disease progression [10,11], the mechanisms remain unclear. Maintaining adequate intestinal IgA production may prevent intestinal inflammation as well as the deterioration of the intestinal environment associated with metabolic syndrome [12]. Thus, elucidating the interplay between intestinal immunity and diabetes should contribute to disease prevention and symptom alleviation.

Dietary factors are believed to play an important role in the onset of diabetes. Feeding standard mice a high-fat diet (HFD) also induces symptoms of type 2 diabetes, such as obesity and hyperglycemia [13]. Certain fatty acids in the diet directly act on intestinal T cells, regulating their proliferation and differentiation, suggesting that dietary lipids may influence the intestinal immune system [14]. In the small intestine of HFD-fed mice, the expression of inflammation-related genes is altered and a reduction in IgA-producing B cells occurs [[15], [16], [17]]. Such effects in the intestine are associated with the induction of insulin resistance and the development of diabetes.

The involvement of various genes in the onset of diabetes has been suggested. KK-A^y^ mice are widely used as a genetic diabetes model characterized by excessive insulin secretion and obesity. The KK (Kuo Kondo) mouse, a polygenic mutation model of type 2 diabetes, exhibits mild diabetic symptoms, whereas the heterozygous KK-A^y^ mouse represents severe obesity and diabetes [[18], [19], [20]]. Changes in the intestinal immune system of KK-A^y^ mice have not been previously reported; however, abnormalities, such as a reduction in NKT cells in the liver [21] and dysbiosis of the gut microbiota [22] have been observed in KK-A^y^ mice, suggesting that changes in the intestinal immune system likely occur.

We conducted this study to elucidate the impact of diabetes on the intestinal antibody repertoire, using two different diabetes models. Changes in the intestinal immune system and gut environment have been observed in various diabetic mouse models, and it is becoming increasingly clear that these changes are related to its pathophysiology. The HFD and KK-A^y^ mouse models induce obesity and diabetic symptoms; however, the processes leading to their onset are different. Therefore, by comparing changes in the intestinal antibody repertoire between these two models, it is possible to determine whether these changes occur as a result of diabetes symptoms following the onset of diabetes, or whether they are influenced by dietary and genetic factors that occur before its onset. We hypothesized that evaluating the antibody repertoire will lead to the improved diagnosis of diabetes, whereas modulating the repertoire may contribute to treatment and prevention. Therefore, in the present study, we evaluated the intestinal IgA repertoire in HFD models and genetically diabetic KK-A^y^ mice to determine whether there are similarities between the two groups.

## 2 Materials and methods

### 2.1 Reagents

Protease inhibitor cocktail, skim milk, and LabAssay Glucose kit were obtained from Wako (Osaka, Japan). Mouse IgA or IgG enzyme-linked immunosorbent assay (ELISA) Antibody Pair Kit was obtained from STEMCELL technologies. RNAlater was purchased from Thermo Fisher Scientific.

### 2.2 Mice

All experiments were conducted according to the guidelines of the Animal Usage Committee of the Faculty of Agriculture, Meijo University and were approved by the ethics committee (Permission No. 2024PE12 and 2024AE13). Four-week-old male C57BL/6JJcl and KK-A^y^/TaJcl mice were obtained from Clea Japan and housed in the animal care facility under controlled temperature and humidity conditions with a 12-h light/dark cycle. C57BL/6J mice were randomly assigned into the following groups: one group was fed a control diet of D12492 (D12450J, 10 % kcal fat, Research Diets), and another group was fed an HFD (D12492, 60 % kcal fat, Research Diets) starting at five weeks of age for 12 weeks. Meanwhile, KK-A^y^/TaJcl mice were fed a control diet containing 10 % kcal fat (D12450J, Research Diets) during the same period. Body weights were assessed every other week.

### 2.3 Collection of biological samples

For urine and feces collection, 14-week-old mice were individually housed in metabolic cages for 18 h with access to food and water ad libitum. Urine samples were obtained and centrifuged at 5000 rpm for 5 min at 4 °C, and the supernatant was aliquoted and stored at −80 °C until use. Fecal pellets were collected, weighed, and added (100 mg/mL) to sterile phosphate-buffered saline (PBS) containing 5 % fetal calf serum and 0.1 % sodium azide. After incubation for 1 h at 4 °C, the pellets were homogenized, and particulate debris was removed by centrifugation for 10 min at 13,000×*g*. The supernatants were collected, and stored frozen at −70 °C until use.

Twelve weeks after the initiation of feeding, mice were euthanized using isoflurane anesthesia. Blood was collected, and the small intestine was rapidly excised. Blood samples were allowed to clot for 30 min at room temperature, then serum was obtained by centrifugation at 3500 rpm for 10 min and stored at −80 °C. The small intestine was removed and washed, and 5 mL of PBS (pH 7.4) containing a protease inhibitor cocktail was passed through the intestine to collect the intestinal fluid. The washout material was centrifuged at 5000 rpm for 20 min at 4 °C, and the supernatant was harvested.

### 2.4 Electrophoresis and Western blot analysis

Intestinal fluid (final dilution 1:1.8) was mixed with Laemmli sample buffer heated at 80 °C for 10 min, and 30 μL of each sample was placed in 10 % acrylamide gels. Intestinal fluid proteins were separated using 10 % polyacrylamide gels on a PAGE apparatus (ATTO, model AE-6500, Tokyo, Japan) via a Power PAC 300 (Bio-Rad). After electrophoresis, the proteins were transferred onto a PVDF membrane (0.45 nm, Wako) by using Transblot SD semidry blotting apparatus (Bio-Rad). PVDF membranes were blocked with 5 % skim milk in Tris-buffered saline with 0.05 % Tween 20 (TTBS) for 1 h at room temperature followed by washing with TTBS and incubation with horseradish peroxidase-labeled anti-IgA antibody (Abcam) at 1:2500 dilution for 1 h at room temperature. Immunolabelled proteins were visualized using a chemiluminescence method (ImmunStar HRP Substrate Kit, Bio-Rad Laboratories Inc.) and detected using ImageQuant LAS 500 (GE Healthcare). Visualized blots were digitized using the ImageQuant TL software (GE Healthcare).

### 2.5 ELISA

Intestinal fluid and fecal IgG or IgA levels were evaluated using the ELISA Antibody Pair Kit according to the manufacturer's protocol. Plates were read using at 405 nm, and sample values were quantified by comparing them to a standard curve.

### 2.6 Repertoire analysis

For repertoire analysis, approximately 100 mg of the small intestine was sectioned into approximately 5-mm cubes immediately after sample collection and immersed in RNAlater. Samples were stored overnight at 4 °C, removed from the liquid, and stored at −80 °C until further analysis. B-cell receptor (BCR) repertoire analysis of the small intestine was performed by Repertoire Genesis Inc. (Osaka, Japan), and BCR diversity was evaluated.

### 2.7 Statistical analysis

The data are presented as mean ± the standard deviation (SD) where indicated. The results were analyzed by a one-way ANOVA followed by Dunnett's or Tukey's post hoc test. GraphPad Prism 8 (GraphPad Software) was used for all analysis. Differences were considered statistically significant at *p* < 0.05.

## 3 Results

### 3.1 Physiological changes in diabetes-induced mice

**Fig. 1:**
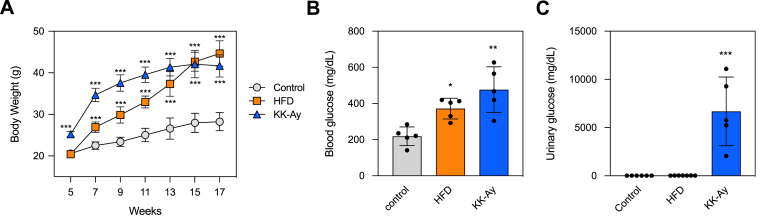
**Characteristics of HFD and KK-A**^**y**^**diabetic mice.** (A) Total body weight (n = 6–7). (B) Serum glucose and (C) urinary glucose levels of non-diabetic control mice, HFD, and KK-A^y^ mice (n = 5–7), respectively. Serum was collected at 17 weeks of age, and urine was collected at 14 weeks of age. Each symbol represents a mouse. Differences were analyzed by Dunnett's test (A) and Tukey's test (B and C). ∗, *p* < 0.05; ∗∗, *p* < 0.01; ∗∗∗, *p* < 0.001; versus control.

To confirm changes in intestinal antibody production related with diabetes, we induced diabetes using an HFD and KK-A^y^ mice. Starting at 5 weeks, the control and KK-A^y^ mice were fed a regular diet, while mice in the HFD group were fed a diet containing 60 % kcal fat. After 12 weeks, the weight of HFD and KK-A^y^ mice was significantly higher than control mice (Fig. 1A). Additionally, it was found that the average body weight of the HFD group had increased more. Furthermore, serum glucose levels in HFD and KK-A^y^ mice were higher than those in control mice (Fig. 1B). Meanwhile, urinary glucose levels at 14 weeks significantly increased in KK-A^y^ mice, whereas urine glucose was not detected in the control and HFD mice (Fig. 1C).

### 3.2 Production of gut antibodies is increased in diabetes-induced mice

**Fig. 2:**
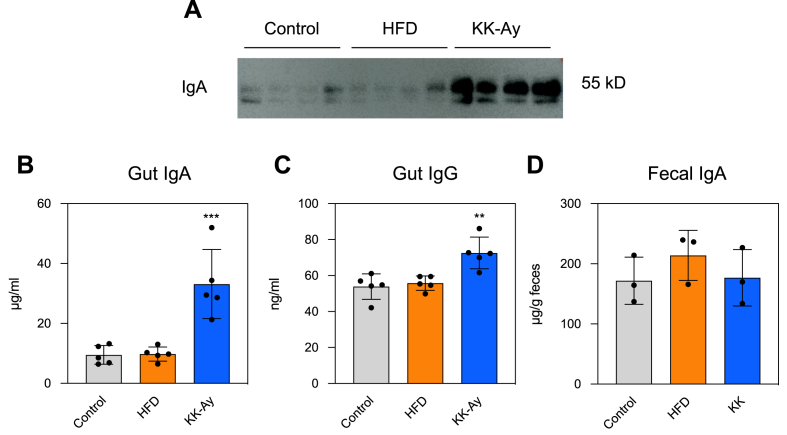
**Determination of immunoglobulin levels in the small intestine.** (A) Western blotting of intestinal fluid IgA (n = 4) at 17 weeks. Intestinal fluid was subjected to SDS–PAGE. Antibodies were visualized by Western blotting using anti-IgA antibodies. (B) Gut IgA and (C) IgG, and (D) fecal IgA levels at 17 weeks (gut) or 14 weeks (feces) were determined by ELISA. Each symbol represents a mouse. Differences were analyzed by Tukey's test. ∗∗, *p* < 0.01; ∗∗∗, *p* < 0.001; versus Control.

Western blotting and ELISA confirmed a significant increase in IgA levels in KK-A^y^ mice (Fig. 2A and B). Due to extremely low levels, IgG levels were not detected by Western blotting (data not shown). However, ELISA showed that IgG levels significantly increased only in KK-A^y^ mice (Fig. 2C). IgA detection was performed on fecal samples collected at 14 weeks, and no significant differences were observed among the three groups (Fig. 2D).

### 3.3 Changes in gut antibodies produced in diabetic mice

**Fig. 3:**
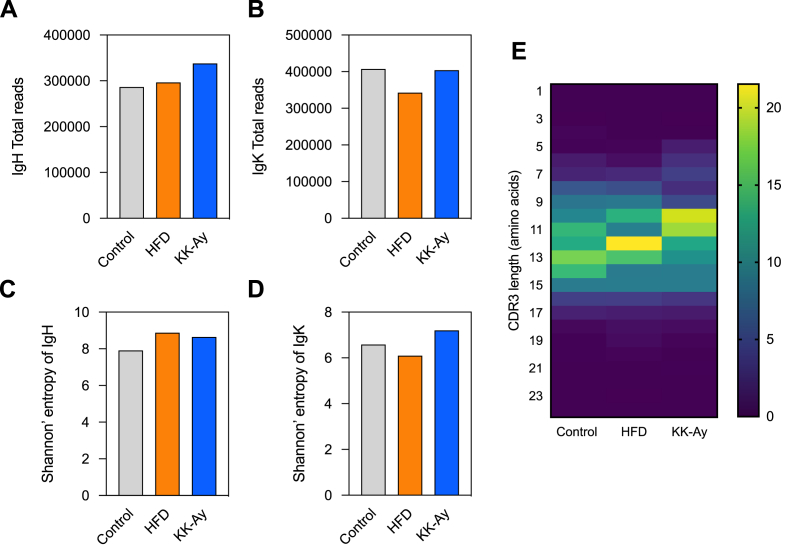
**Clonal distributions of intestinal B-cell repertoires.** The number of CDR3 sequences of (A) IgA heavy chain and (B) kappa light chain. Shannon's entropy of BCR repertoire in the (C) IgH region of IgA and (D) IgK region. (E) The distribution of CDR3 lengths of IgH in Control, HFD, and KK-A^y^ mice. Heatmaps were generated from sequences with ≥10 reads.

Increased intestinal antibody production was noted in KK-A^y^ mice. Thus, to confirm the changes in antibody sequence, repertoire analysis was conducted. In each group, small intestine tissues were collected at 17 weeks old. BCR repertoire analysis comprehensively decoded the immunoglobulin heavy chain (IgH) sequences of IgA and the immunoglobulin kappa light chain (IgK) sequences of all isotypes. The total number of antibody sequence reads was approximately 300,000 for IgH and 400,000 for IgK (Fig. 3A and B). When the diversity of the antibody repertoire was compared using Shannon entropy, an increasing trend was observed for IgH in HFD and KK-A^y^ mice (Fig. 3C). Meanwhile, for IgK, decreasing and increasing trends were observed in HFD mice and KK-A^y^ mice, respectively (Fig. 3D). The distribution of the CDR3 length of IgH was also compared (Fig. 3E) and the averages were as follows: control, 13.98; HFD, 13.93; and KK-A^y^, 13.51. Both HFD and KK-A^y^ mice had shorter CDR3 lengths of IgH compared to control mice.

### 3.4 Significant changes in the antibody repertoire in diabetic mice

**Fig. 4:**
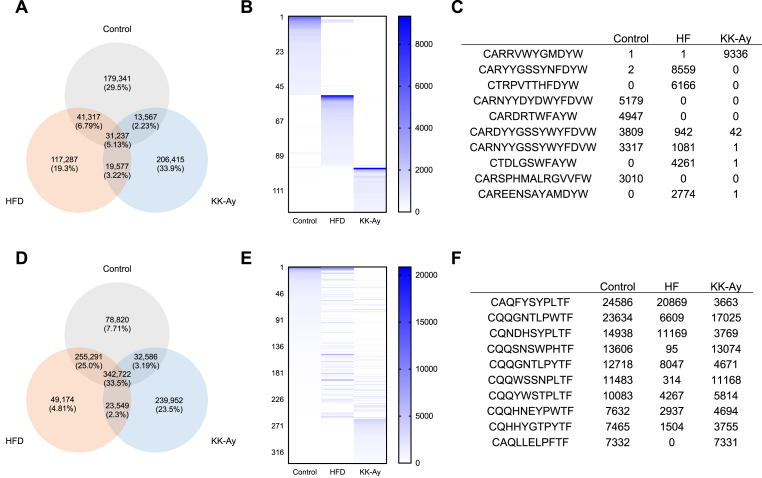
**Clonal overlap of CDR3 sequences in small intestinal antibodies among the three groups.** Number of overlapped sequences of (A) IgA heavy chain and (D) kappa light chain. Comparison of high-frequency CDR3 sequences of (B) IgH and (E) IgK in each group. Heat map indicates the distribution of highly read IgA IgH sequences. The y-axis shows each (B) 124 and (E) 334 sequences that were detected with ≥500 reads. Ten sequences each for (C) IgH and (F) IgK with the highest total number of reads.

Next, we investigated the extent of overlap in antibody sequences in the antibody repertoire among the three groups (Fig. 4A). Our results showed that a significant proportion of sequences were independent across all three groups, with only few sequences common to two or all groups. The distribution of sequences with reads ≥500 (Fig. 4B) and the top 10 sequences with the highest number of reads (Fig. 4C) in IgH are illustrated, confirming that HFD and KK-A^y^ mice possess sequences that are read with high frequency, which were almost absent in control mice. Similarly, we also showed the proportion for IgK (Fig. 4D). The results showed that the sequences common to all three groups were the most abundant, followed by those common between the control and HFD mice, and those unique to KK-A^y^ mice. When examining the sequence distribution of IgK with reads of ≥500 (Fig. 4E) and the top 10 most read sequences (Fig. 4F), it matched the results from Fig. 4D. However, unlike IgH, there were few sequences common among the three groups.

## 4 Discussion

In this study, several experiments were conducted using HFD and KK-A^y^ mice, and various changes in the intestinal antibodies associated with diabetes were confirmed. In HFD mice, diabetes was confirmed by elevated blood glucose levels after 12 weeks of feeding (Fig. 1A and B); however, antibody production remained unchanged (Fig. 2A–C). It was reported that the IgA level in the distal small intestine was not affected in HFD mice, similar to our results [17]. Meanwhile, IgA production in HFD mice was reportedly decreased in the small intestine [23,24]. Although various studies have conflicting findings, our results showed that there was no change in IgA production in HFD mice. By comparing the effects of HFD with varying fat content or long- and short-term HFD feeding on changes in diabetes symptoms and the intestinal immune system, it is possible to gain deeper insights.

In KK-A^y^ mice, the production of intestinal IgA and IgG significantly increased, and that mucosal immunity was activated (Fig. 2A–C). Mice transplanted with feces from diabetic patients showed enhanced intestinal IgA production [25]. Because changes in gut microbiota are also observed in KK-A^y^ mice [22], they may be involved in antibody production. However, the amount of fecal IgA did not increase, which contrasted with that in the intestine (Fig. 2D). When collecting intestinal fluid, IgA bound to microbes is removed by centrifugation, suggesting a potential increase in IgA binding to microbes. The reason why an increase in IgA production was only observed in KK-Ay mice and not in HFD mice remains unclear. KK-A^y^ mice exhibited more severe diabetes than HFD mice as urinary glucose was detected only in them (Fig. 1C). The difference in the effect on IgA production between the HFD and KK-A^y^ mice may be attributed to the severity of the symptoms.

This study revealed that the production and CDR sequences of intestinal IgA were influenced by diabetes but varied depending on the diabetic model. We conducted comparative analysis of the antibody sequences of IgH and IgK produced in the small intestine. CDR3 of IgH, which is important in antigen binding, had greater diversity in diabetic mice than in control mice (Fig. 3C), and the amino acid length of CDR3 was shorter (Fig. 3E). Acquired antibodies reportedly have shorter CDR3 [26], and that T cell-dependent antibody production likely occurs against an antigen in two types of diabetic mice, leading to different antibody production. Furthermore, the antibody repertoire has significant variation in IgH (Fig. 4A–C), with very few sequences that were common across two or three groups. Hence, the IgA repertoire is completely different in the three groups. We expected an increase in a common antibody sequence in HFD and KK-A^y^ mice, which could be considered specific to diabetic mice, but such sequence was not confirmed in this study (Fig. 4A–C).

In HFD mice, although no changes in the quantity of IgA were observed, significant fluctuations in the antibody sequences and specificity were evident. The antibody repertoire is reportedly altered in HFD-fed mice [27]; however, the mechanisms behind this phenomenon remain unclear. The change in the IgH repertoire differed between HFD and KK-A^y^ mice, suggesting the existence of a factor other than diabetes. In HFD mice, excessive fat feeding is expected to increase the levels of lipid-derived antigens and directly induce immune response. Furthermore, intestinal inflammation was reportedly facilitated by an HFD, resulting in increased intestinal epithelium permeability [28]. This may contribute to the augmentation of antigen production or the creation of a more inducible environment for immune system stimulation.

During the increase in IgA production in KK-A^y^ mice (Fig. 2A and C), it is believed that not only the activation of IgA production but also changes in the types of IgA produced are likely occurring. The change in antibody specificity suggests that some antigenic molecules are increased in the intestine. Because the KK-A^y^ mice were administrated a regular diet, it is likely that metabolites from the intestinal cells or gut microbiota are involved. Although it is likely that intestinal inflammation also occurs in KK-A^y^ mice [29,30] as their diet is identical to that of control mice, the possibility of an increase in lipid antigen levels in the intestines of KK-A^y^ mice is low. This suggests that changes in the antibody repertoire in KK-A^y^ mice are caused by different factors than those affecting HFD mice. There have been many studies using a model in which HFD is administrated to KK-A^y^ mice to induce more severe diabetes [31]. By analyzing the repertoire, it is anticipated that clarifying how the antibody repertoire changes when an HFD is administrated to KK-A^y^ mice, and how it differs from administering an HFD to normal mice or a regular diet to KK-A^y^ mice, will provide insight into the relationship between the repertoire and the pathophysiology.

Changes in IgA production and antibody repertoire are likely related to the gut microbiota. The gut microbiota plays an important role as an antigen for intestinal IgA antibodies [32], and it is also involved when diet affects the immune system. The consumption of a high-protein diet alters the gut microbiota, and its metabolites stimulate intestinal epithelial cells to induce IgA production [23]. In addition, gut microbiota metabolites affect the antibody repertoire [5]. The gut microbiota undergoes significant changes in HFD-fed and KK-A^y^ mice [22,23]. By examining the gut microbiota and its metabolites in HFD and KK-A^y^ mice and by identifying the differences in the antigen profiles mediated by the gut microbiota in both models, it will be possible to identify the mechanisms underlying antibody production. This will provide insight into the factors responsible for the differences observed in the antibody repertoire.

In the present study, the IgK repertoire did not undergo significant changes because of diabetes, unlike the IgH repertoire (Fig. 4D–F). In addition to the absence of the D gene, the IGKV genes used in IgK were highly restricted and its diversity was much smaller compared to that of IgH [33]. The small diversity of this light chain may be involved in avoiding auto-reactivity, among other potential functions. This is considered an important finding that IgK does not change when the diversity of IgH is altered in diabetes. Identifying the reason for the lack of diversity in IgK may be useful for understanding the factors that contribute to the establishment of IgH diversity; therefore, further studies are needed.

The repertoire analysis in the present study has several limitations. Intestinal samples were collected from five animals in each group and analyzed collectively. Therefore, it is not possible to determine whether specific CDR3 sequences are increased in all individuals of the same group or only in specific individuals. Further studies are needed to identify antibodies specific to diabetic mice that experience increased levels uniformly across all individuals. In particular, when examining the functionality of individual antibodies, analysis on a per-animal basis is essential. For example, when conducting a detailed study on specific antibody sequences that increase in diabetic model mice in the future, a repertoire analysis should be conducted on each mouse, and it will be necessary to demonstrate that the antibody increases in all mice.

Future studies should investigate how sex differences affect the antibody repertoire. Both HFD and KK-A^y^ mice develop diabetes regardless of sex, but it has been reported that the symptoms are influenced by sex differences. In mice fed an HFD, males exhibit more prominent hyperinsulinemia and inflammatory states, whereas females show a suppression of symptoms compared with males [34]. In KK-A^y^ mice, although diabetes symptoms were observed in both sexes, sex differences have been identified in aspects such as bone strength reduction [35]. Currently, the mechanisms by which diabetes alters the antibody repertoire remain unclear. Therefore, by conducting a detailed analysis of the differences in repertoire changes based on sex, the relationship is expected to become clearer. Therefore, we believe it is necessary to evaluate the impact of sex differences in subsequent studies.

In applying the changes in the antibody repertoire identified in this study to therapy, it is important to clarify which specificities and functions of antibodies are increased in diabetic mice. Computer simulations are making it increasingly possible to infer antigens from antibody sequences [36,37] Further characterization of the antibodies induced by diabetes, as revealed in the present study, may be useful in understanding their involvement in the onset or progression of the disease. In particular, although the antibody sequences that are increased in diabetes have been identified, the antigens and functionalities of these individual antibodies are unclear. At the stage of identifying the antigens associated with these antibodies, computer simulations are expected to be effective. In both HFD and KK-A^y^ mice, antibody production with different sequences was observed. However, functional analysis may suggest that despite the differences in sequences, these antibodies may have the same specificity or functionality. Furthermore, by elucidating the characteristics of diabetes-induced antibodies from their amino acid frequency, hydrophobicity, and 3D structure, it may be possible to identify the characteristics of the induced antibodies, the features of the putative antigens, and the mechanisms driving their production, without the need for individual analysis.

This study suggests that the effect on mucosal immunity varies depending on the pathogenesis of diabetes, and it is anticipated that factors that contribute to the progression of diabetes, such as diet and genetic factors, may be involved. Future studies should determine how the changes in the antibody repertoire in the intestine are related to the onset and progress of diabetes and gain insights that will help in the prevention and treatment of the disease. By analyzing individual antibodies that increase in diabetes, it may be possible to determine whether each antibody contributes to the worsening of the disease or has a preventive effect. Furthermore, if it is discovered that certain food components or metabolites trigger the production of antibodies that are associated with the worsening of diabetes, removing these substances may inhibit the production of specific antibodies and disease progression. As further insights are gained into the qualitative changes in antibodies, this may result in more effective prevention and treatment strategies. These findings may contribute to the prevention and treatment of the disease by controlling the production of antibodies that are increased in diabetes.

## CRediT authorship contribution statement

**Miho Chikazawa:** Writing – review & editing, Writing – original draft, Visualization, Methodology, Investigation, Funding acquisition, Formal analysis, Data curation, Conceptualization. **Ken-Ichiro Minato:** Writing – review & editing, Investigation, Funding acquisition, Conceptualization.

## Declaration of generative AI and AI-assisted technologies in the writing process

In the process of preparing this manuscript, the authors utilized DeepL, Grammerly, and ChatGPT to improve readability and language.

## Funding

This study was supported by research grants from the 10.13039/501100001700Ministry of Education, Culture, Sports, Science and Technology of Japan (No. 21K05476 to M.C.), the 10.13039/501100011907Mishima Kaiun Memorial Foundation, and the Research Institute of 10.13039/501100009380Meijo University (Promotion of Scientific Research Subsidy).

## Declaration of competing interest

The authors declare the following financial interests/personal relationships which may be considered as potential competing interests:Miho Chikazawa reports financial support was provided by Government of Japan Ministry of Education Culture Sports Science and Technology. Miho Chikazawa reports financial support was provided by Public Interest Foundation 10.13039/501100011907Mishima Kaiun Memorial Foundation. Ken-Ichiro Minato reports financial support was provided by Research Institute of 10.13039/501100009380Meijo University. Miho Chikazawa reports writing assistance was provided by Enago.

## Figures and Tables

**figs1:**